# A Systematic Review of Human Bat Rabies Virus Variant Cases: Evaluating Unprotected Physical Contact with Claws and Teeth in Support of Accurate Risk Assessments

**DOI:** 10.1371/journal.pone.0159443

**Published:** 2016-07-26

**Authors:** Virginia M. Dato, Enzo R. Campagnolo, Jonah Long, Charles E. Rupprecht

**Affiliations:** 1 University of Pittsburgh, School of Medicine, Pittsburgh, PA, United States of America; 2 Pennsylvania Department of Health, Bureau of Epidemiology, Harrisburg, PA, United States of America; 3 Centers for Disease Control and Prevention, Office of Public Health Preparedness and Response, Division of State and Local Readiness, Field Services Branch, Atlanta, GA, United States of America; 4 The Wistar Institute, Philadelphia, PA, United States of America; CSIRO, AUSTRALIA

## Abstract

In the United States and Canada, the most recent documented cases of rabies have been attributed to bat rabies viruses (RABV). We undertook this systematic review in an effort to summarize and enhance understanding of the risk of infection for individuals who have been potentially exposed to a suspect or confirmed rabid bat. United States rabies surveillance summaries documented a total of 41 human bat-rabies virus variant verified non-transplant cases between 1990 and 2015. All cases were fatal. Seven (17.1%) of 41 cases reported a bite from a bat. Ten (24.3%) cases had unprotected physical contact (UPC); these included seven cases that had a bat land or crawl on them (contact with claws) and one case that touched a bat’s teeth. Seven (17.1%) cases had probable UPC. Insectivorous bat teeth are extremely sharp and highly efficient for predation upon arthropod prey. Bats also have sharp claws on the end of their thumbs and feet. One of the most common bat RABV variants has an ability to replicate in non-neural cells. Questioning individuals about unprotected contact with bat teeth and claws (including a bat landing or crawling on a person) may help identify additional exposures.

## Introduction

In the United States and Canada, most of the documented cases of rabies in humans during the past decade have been attributed to bat rabies viruses (RABV), in contrast to developing countries where canine rabies predominates [1–5]. As such, 17 of 21 (81.1%) non-organ transplant cases acquired in the United States between 2003 and 2015 were linked epidemiologically or by phylogenetic analysis to exposure to bat RABV [6]. More than 30 different species of bats have been diagnosed with rabies, and major taxa maintain a unique RABV variant [7–11].

Suspected human exposure to rabid bats places a significant economic burden on the public health system. In North America and Western Europe, bats account for 5–10% of the total human rabies post-exposure prophylaxis (PEP) burden, since the first rabid insectivorous bat was discovered in Florida during 1953 [12–14]. One recent California study reported that the mean total cost of a suspected human RABV exposure was $3,688, with direct costs of $2,564 and indirect costs of $1,124 [15].

Accurate cost benefit analysis requires analysis of the risk of transmission in a variety of scenarios [16]. Additionally, the benefits of providing PEP should always be carefully weighed against all risks including the possibility of adverse events [17,18].

Bat RABV variants would be expected to vary in their relative virulence characteristics, especially as related to the basic infectious reproductive number, R0 [19]. Bat species vary greatly in their degree of coloniality, from solitary to the highly gregarious, which may influence transmission dynamics.

We undertook a historical review of confirmed human bat RABV cases in the United States reported from 1990 to 2015, to determine and characterize the most likely exposure. Our objective was to summarize and enhance understanding of the risk of infection for individuals who have been potentially exposed to a suspect or confirmed rabid bat.

## Materials and Methods

**Fig 1:**
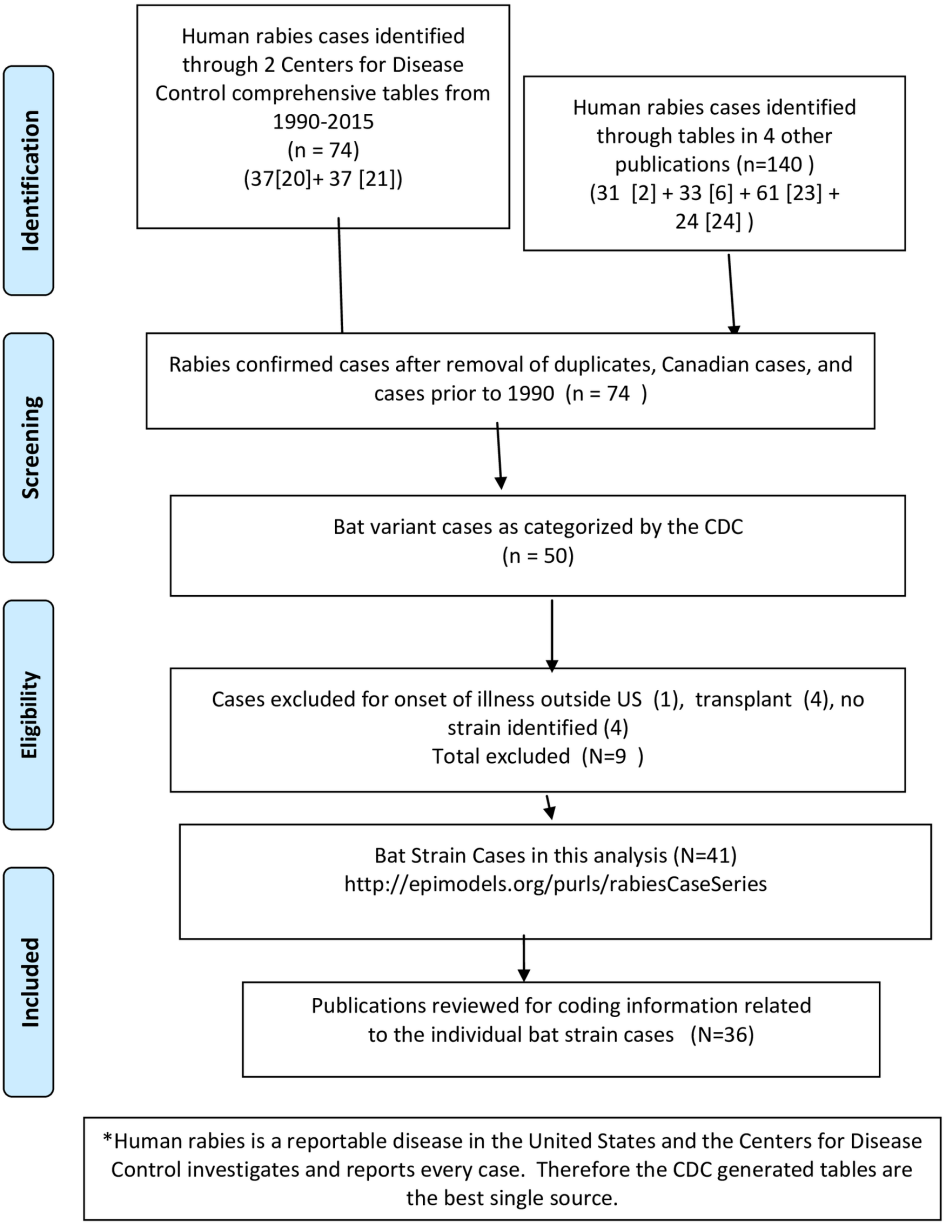
Flow Diagram for Systematic Review of Human Rabies Bat Rabies Virus Variant Cases*. *Adapted from*: Moher D, Liberati A, Tetzlaff J, Altman DG, The PRISMA Group (2009). *P*referred *R*eporting *I*ems for *S*ystematic Reviews and *M*eta-*A*nalyses: The PRISMA Statement. PLoS Med 6(7): e1000097. doi:10.1371/journal.pmed1000097 **For more information, visit** www.prisma-statement.org.

Human cases of rabies reported in the United States between 1990 and 2015, are listed in published rabies surveillance summaries. Two prior summaries [6,20] contain all reported cases by circumstance of exposure and RABV variants for all 25 years of this review (Fig 1). Other published series [2,21–24] did not reveal additional cases. The identified cases of human rabies were augmented further by review of published individual case histories [2,25–57].

Cases were categorized by the first author of this study, and then reviewed by at least one other co-author. Consensus was reached on any differences by the co-authors. Although some case investigators had been contacted, only published data were used for the final classifications. For inclusion in the study, a case had to be verified as caused by a bat RABV variant, disease onset must have been in the United States and the patient must not have undergone a recent organ transplant.

Cases were classified initially as to whether there was a report of a bat bite or scratch, some other form of unprotected physical contact (UPC), a probable unprotected physical contact (PUPC), or as unknown. Cases caused through transplantation of organs or tissues were excluded from this analysis. A case was classified as UPC if there was definite physical contact reported, and gloves, or other consistent means of personal protection, were not mentioned in the interview. A case was classified as PUPC if the individual was known to have caught, or removed, a bat, but a reliable interview with the patient was not available to determine, with certainty, that the individual had UPC with a bat. All other cases, where contact could not be determined, or was not reported, were classified as unknown.

In addition to a thorough review of the cases, each individual case report was evaluated for additional variables including whether the case was reported to have removed a bat from a home, who the informants were for contact with a bat, when the diagnosis for rabies was first considered seriously (based upon the ordering of diagnostic tests and consultation with public health professionals), and how many human contacts of the case were given PEP. Contacts were considered to be healthcare-associated if they resulted from any component of the healthcare response to illness, including initial visits, emergency responders, hospital staff and mortuary services. Other contacts included family, friends, classmates, co-workers and any other individual who interacted with the patient outside of a job responding to sick or deceased individuals.

To determine if the individual had been sleeping while a bat was in the bedroom, the words bedroom, awakened or sleep, were searched electronically. Cases were coded as ‘yes’ for “bedroom” if a case was reported to have awakened to a bat, or had been sleeping while a bat was present in the same room or immediate vicinity. ‘No’ included cases where a location other than a bedroom was specified for exposures that did not involve awakening from sleep. Unknown included locations not specified, including a home, where the exposure may or may not have been in a bedroom.

Cases with UPC were also reviewed to determine if exposure to claws or teeth was documented. If a bat had either landed or crawled on a person, and clothing or other barriers were not mentioned, a UPC to claws or teeth was presumed.

Given the inherent bias in the utilization of historical data where individuals often could not be interviewed prior to death and the lack of appropriate controls, only descriptive statistics were utilized.

## Results

A review of the peer-reviewed literature identified 41 published, confirmed non-transplant human rabies cases associated with bat RABV in the United States from 1990 to 2015. The full case series with demographic, pathogen and descriptive source information is available at http://epimodels.org/purls/rabiesCaseSeries [58]. Selected variables are available in Table 1. All cases were fatal. Only seven (17.1%) cases reported known perceived ‘bites’ from a bat (Table 2). No cases reported scratches. Ten (24.4%) reported cases had UPC, and seven (17.1%) reported cases had PUPC with a bat. Seventeen (41.5%) of the 41 bat RABV cases had an unknown exposure to a bat. No case was known to have been previously vaccinated with rabies vaccine. The most common RABV variants were 26 Ln/Ps-related (63.4%) and 10 Tb-related (24.4%) cases. No associations were noted between exposure type and RABV variants.

**Table 1:** Confirmed Cases of Non-Transplant Human Rabies attributed to a Bat RABV, United States, 1990 to 2015).

Case	Year/ State^a^	Age/Sex	Bat Contact Type^b^ /Bed Room^c^ /Removed^d^^e^	Informant/Time Diagnosis Seriously considered	Virus^e^	Human Contacts Given PEP Total/Healthcare^f^/Other	Ref
1	1990/TX	22/M	Bite/No/No	Friend/Antemortem	Tb	67/Unspecified/Unspecified	[25]
2	1991/AR	29/M	UPC/Unk/Yes	Friend/Antemortem	Ln/Ps	99/81/18	[26]
3	1991/GA	27/F	Unk/Unk/Unk	Family &Friends/Antemortem	Ln/Ps	Unspecified	[26]
4	1993/NY	11/F	Unk/Unk/Unk	Unspecified/Postmortem	Ln/Ps	55/44/11	[27]
5	1993/TX	82/M	Unk/Unk/Unk	Patient & Family/ Antemortem	Ln/Ps	73/57/16	[28]
6	1994/CA	44/M	Unk/Unk/Unk	Family/Postmortem	Ln/Ps	26/25/1	[29]
7	1994/AL	24/F	PUPC/No/Yes	Unspecified/Postmortem	Tb	99/85/11	[31]
8	1994/WV	41/M	UPC/No/Unk	Friend & Family/Antemortem	Ln/Ps	48/35/13	[30]
9	1994/TN	42/F	Unk/Unk/Unk	Patient/Antemortem	Ln/Ps	47/35/12	[31]
10	1995/WA	4/F	Unk/Yes/No	Family/Antemortem	Msp	72/16/56	[32]
11	1995/CA	27/M	UPC/No/Unk	Family/Antemortem	Tb	40878	[33]
12	1995/CT	13/F	Unk/Unk/No	Family/Antemortem	Msp	83/46/37	[34]
13	1995/CA	74/M	PUPC/Unk/Unk	Family/Postmortem	Ln/Ps	76/72/4	[33]
14	1996/KY	42/F	Unk/Unk/Unk	Patient & Family/Antemortem	Ln/Ps	87/82/5	[35]
15	1996/MT	49/M	Unk/Unk/Unk	Patient & Family/Antemortem	Ln/Ps	26/23/3	[35]
16	1997/MT	65/M	PUPC/Yes/Yes	Family/Postmortem	Ln/Ps	60/58/2	[36]
17	1997/WA	64/M	Unk/Unk/Unk	Family/Postmortem	Ef	55/54/1	[36]
18	1997/NJ	32/M	PUPC/No/Yes	Patient & Family/Antemortem	Ln/Ps	50/42/8	[37]
19	1997/TX	71/M	UPC/Yes/Yes	Patient &Family/Antemortem	Ln/Ps	46/42/4	[37]
20	1998/VA	29/M	Unk/Unk/Unk	Family, Friends & Co-workers/Antemortem	Ln/PS	48/16/3	[38]
21	2000/CA	49/M	PUPC/Unk/Yes	Patient & Family/Antemortem	Tb	Unspecified	[39]
22	2000/GA	26/M	UPC/Yes/Unk	Co-workers/Unspecified	Tb	Unspecified	[39]
23	2000/MN	47/M	Bite/Yes/Unk	Family &Friends/Unspecified	Ln/Ps	Unspecified	[39]
24	2000/WI	69/M	UPC/Unk/Yes	Patient & Family/Antemortem	Ln/Ps	Unspecified	[39]
25	2002/CA	28/M	Unk/Unk/Yes (killed bat in home)	Family/Antemortem	Tb	46/28/18	[41]
26	2002/IA	20/M	Unk/Unk/Unk	Family & Friends/Antemortem	Ln/Ps	124/71/53	[40]
27	2002/TN	13/M	PUPC/No/Unk	Family/Antemortem	Ln/Ps	Unspecified	[42]
28	2003/CA	66/M	Bite/Yes/Yes	Patient/Antemortem	Ln/Ps	38140	[43]
29	2004/AR	20/M	Bite/Unk/Unk	Others/Postmortem	Tb	Unspecified	[44] [59] [45]
30	2006/TX	16/M	UPC/Yes/Unk	Family & Acquaintances/Antemortem	Tb	Unspecified	[46]
31	2006/IN	10/F	Bite/Yes/No	Patient &Family/ Antemortem	Ln/Ps	66/27/38	[47]
32	2008/Ca	16/M	Unk/Unk/Unk	Family & Friends/Postmortem	Tb-related	20/4/16	[48]
33	2008/MO	55/M	Bite/Unk/Yes	Patient & Family/Antemortem	Ln/Ps	38108	[49]
34	2009/IN	43/M	Unk/Unk/Unk	Family, Friends & Co-workers/Antemortem	Ln/Ps	18/14/4	[50]
35	2009/MI	55/M	UPC/Yes/Yes -	Family (relative)/Antemortem	Ln	18/6/12	[51]
36	2010/LA	19/M	Bite/Yes/Unk	Family/Antemortem	Ds	95/68/27	[52]
37	2010/WI	70/M	Unk/Unk/Unk	Family/Antemortem	Ln/Ps	37442	[53]
38	2011/SC	46/F	PUPC/Yes/Yes	Family/Antemortem	Tb	22/18/4	[54]
39	2011/MA	63/M	UPC/Yes/Unk	Family/Antemortem	Msp	14/9/5	[55] [24]
40	2014/MO	52/M	Unk/Unk/Unk	Family, Friends/Antemortem	Ps	16/7/9	[56]
41	2015/WY	77/F	UPC/Yes/No	Family/Antemortem	Ln	26/22/44	[6] [57]

^a^. Year and state at onset of illness.

^b^. UPC = Unprotected Physical Contact; PUPC = Probable Unprotected Physical Contact. Full text for all bat contact exposures is available at: http://epimodels.org/purls/rabiesCaseSeries and in the references.

^c^. Bedroom: Yes = includes cases reported to have awakened to a bat, or had been sleeping while a bat was present in the same room or immediate vicinity. No = includes cases where a location other than a bedroom was specified for exposures that did not involve awakening from sleep. Unknown = includes locations not specified.

^d^. Did the individual remove the bat from a room or house? If the individual killed the bat, this was considered as removing it.

^e^. The RABV variant as reported by the CDC: Ln = *Lasionycteris noctivagans* (the silver-haired bat); Ps = *Perimyotis subflavus* (the eastern tri-colored bat); Msp = *Myotis* species (the mouse-eared bats); Tb = *Tadarida brasiliensis* (the Mexican free-tailed bat); Ds = *Desmodus rotundus* (the common vampire bat); Ef = *Eptesicus fuscus* (the big brown bat)

^f^. Health care includes emergency and mortuary response.

**Table 2:** Selected characteristics related to non-transplant cases of bat RABV reported in humans in the United States, 1990–2015.

Bat Contact Category	Number (%)	Mean age (std dev)	Number Male (%)	Mean Non Health Care Contacts (std dev)	Bed room (%)	Bat Removal (%)	Post-mortem Diagnosis (%)
Bite	**7 (17)**	**34.1 (22)**	**6 (86)**	**18.3 (17.1)**	**4 (57)**	**2 (29)**	**1 (14)**
Unprotected physical contact (UPC)	**10 (24)**	**47.4 (22.2)**	**9 (90)**	**9.6 (5.4)**	**6 (60)**	**4 (40)**	**0 (0)**
Probable unprotected physical contact (PUPC)	**7 (17)**	**43.3 (21.9)**	**5 (71)**	**5.8 (3.6)**	**2 (29)**	**5 (71)**	**3 (43)**
Unknown	**17 (42)**	**37.4 (21.9)**	**11 (65)**	**15.4 (17.8)**	**1 (6)**	**1 (6)**	**4 (29)**
Total Cases	**41 (100)**	**40.3 (21.6)**	**31 (76)**	**13.3 (14.3)**	**13 (32)**	**12 (29)**	**8 (20)**

The UPC cases included eight with known or presumed teeth or claw contact with a bat. These cases included a 41 year-old male who had examined a bat “by opening its mouth and feeling the teeth” [30], as well cases where the exposure was attributed to a bat landing or crawling on a person’s face [26,46], chest [33], shoulder [37], arm [51], neck [57], and an unspecified location [39].

Thirteen (31.7%) cases also had a history of a presence of a bat in the room or immediate vicinity in which they were sleeping (Table 2). Of these thirteen cases, four had known bites from a bat [39], [39], [47], [52], six had UPC with a bat [2][58][46][37][51][39][57] and two had probable UPC with a bat [36], [54]. Five cases also had a history of removing a bat from their dwelling [54][43][37][36][51]. Only one case, a four year-old child (1995 WA) [32] had a potential ‘sleep’ exposure to a bat, but with no exposure that met the definition of a bite, UPC, or probable UPC. This four year-old had denied a bite, or any contact with the bat, but the family was still concerned enough to look for, but did not find, any evidence of a bite on the child.

In 16 (39.0%) of the cases reported, the exposure was noted as unknown with no documentation of a bat in the bedroom. Furthermore, none of these individuals were likely to have been directly interviewed about UPC including claw or teeth contact with a bat prior to their death.

## Discussion

Bats differ from other mammalian rabies reservoir species, in their distinct anatomy, physiology, behavior, and ecology. Notably, all bats fly, and many species can locate prey and avoid obstacles by means of their sonar (i.e., echo-location). When a bat is impaired neurologically, as would be the case with RABV infection, an affected bat cannot rely accurately on sonar for flight navigation, and in a paretic state, a bat is at an increased risk of ataxia, grounding, or landing upon a human or domestic animal, which an otherwise healthy bat would avoid [60] [61].

**Fig 2:**
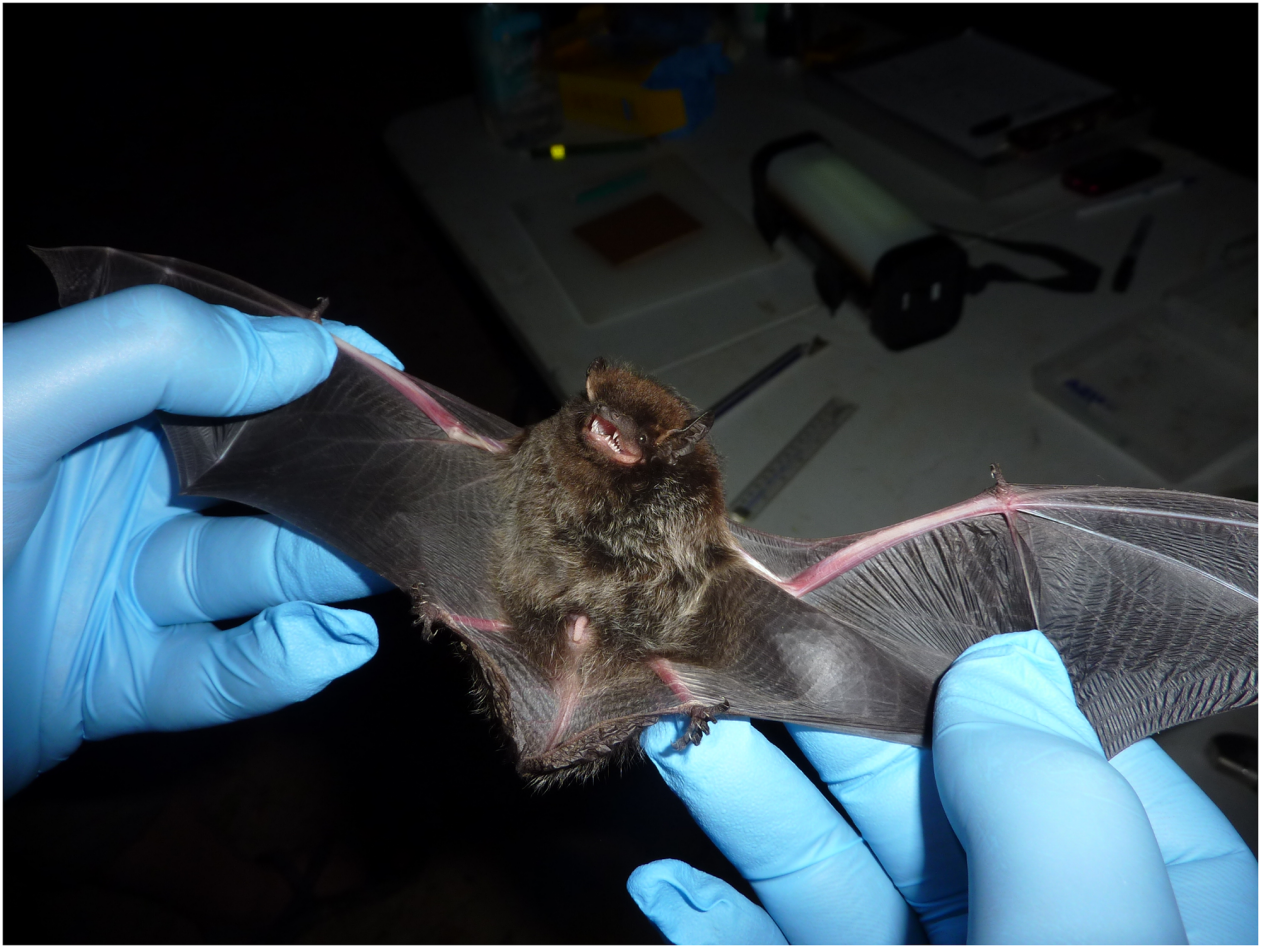
Silver-haired bat with tiny teeth, extended thumb claw at the top of the wing, and clawed feet. Source: "Silver-haired bat" by Larisa Bishop-Boros—Own work. Licensed under CC BY-SA 3.0 via Commons—https://commons.wikimedia.org/wiki/File:Silver-haired_bat.JPG#/media/File:Silver-haired_bat.JPG.

When considering human exposures to bats in the United States and Canada, certain anatomical attributes of bats are relevant. Insectivorous bat teeth are small, needle-like, sharp and highly efficient for the capture, killing, and consumption of arthropod prey. Skin on a bat’s wing covers all the fingers except a short thumb, which is left free (Fig 2). A sharp claw on the end of the thumb forms a functional ‘hook’ at the top of the wing, which aids in grooming, grasping, and related functions (Fig 2). When the wings are folded, the bat uses its claws to climb over surfaces. The skin connecting the fingers is also attached near the bat's clawed feet. Similar to human hands, a bat's feet can turn inward, which enables them to grasp objects. Such sharp claws allow bats to hook securely onto surfaces, either when they land or roost on surfaces such as a wall or on the ceiling of caves. The claws are sharp and strong enough to support the bat's entire weight during rest, even during daily torpor or seasonal hibernation, for hours to months. The presence of virus-laden saliva on bat claws and wings, most likely deposited through grooming, or if rabid, via increased salivation during the moribund stages of infection, is one plausible explanation how RABV may be transferred, by means other than an identified bite, through either a superficial skin piercing, a prick, or a scratch when a rabid bat lands upon, or collides accidentally into an individual, during the course of a bat’s encephalitis.

In this review, human cases with UPC with bats outnumbered cases with known bites, and included individuals with contact with claws and teeth that were not reported as bites or scratches. The above-mentioned types of human exposure to rabid bats are consistent with what is known about bat anatomy, rabid animal behavior, and means by which contact with a rabid bat could lead to human exposures via transdermal or mucosal means. All of the cases reported in this review with UPC had opportunities for exposure to bat saliva, for example, through teeth or claws, even if frank lesions were not recognized.

The 17 documented human cases of RABV associated with bats, reported with no known bat bites, definite, or probable UPC warrant further assessments. Only one case had a bat reported in the bedroom (a 4-year old child) [32]. One could pose the question whether these particular cases could have been attributed to unrecognized bats in the bedroom while the individual was sleeping. All adults finding a bat in the bedroom also had definite or probable UPC. In many of these cases, the contact consisted of removing the bat from the bedroom. In other cases, the persons had awakened from contact with a bat.

A history of a bat in a bedroom by itself does not support a high probability of RABV infection. For example, during a 2007 telephone survey conducted in Canada, 0.1% of the surveyed population (n = 36,445) reported finding a bat in a household bedroom for the one-year period of study. The majority (>95%) of these individuals also reported that they did not seek medical assessment, and none were reported to have developed rabies [62]. Similarly, an earlier survey, conducted between 1998 and 1999 of Oregon households, and reported through the Oregon Behavioral Risk Factor Surveillance System (BRFSS) [63] [64, 65], included questions on RABV exposure. Of the 10,804 households responding to the survey, 150 (1.4%) reported finding a bat in their homes within the preceding year and only two reported any household member receiving rabies vaccine after the bat was found. In 40 (27%) of these 150 households, bats were discovered in rooms where someone had been asleep during the previous 12 hours. A total of 72 people were reported by the 40 households as having been asleep in a room when a bat was found. No Oregon resident was reported to develop rabies during this time period [20].

Unprotected physical contact with claws and teeth that may not be perceived as a bite or scratch, and therefore not treated, could be the cause of some of the unknown exposure cases reported. If individuals believe that only visible bleeding or broken skin as occurs with a mammalian carnivore bite causes rabies, minor contact with teeth or claws could easily be ignored. In addition, family and friends may not notice the lesion to question the origin and suggest PEP. The fact that known bites occurred in a minority (7, 17.1%) of rabies cases may simply be due to the fact that a majority of individuals with known bites from rabid bats obtain highly effective PEP.

The 2008 California case (number 32 Table 1) included reports of bites from both a dog and fox but no report of a bat bite. Could this case have been due to spillover from a bat RABV transmitted via a carnivore? Perhaps in theory, but neither of the other suspect animals were shown to have rabies. The presence of two bites may have been simply an indication of a human who had frequent contact with wildlife, including bats. Reports of the two bites were from family and friends, who might more easily notice a bite from a carnivore but could miss a bite, or would not know of UPC from a bat, unless specifically informed.

The fatality case rate for this series was 100%. This was a direct result of the case definition that required that “a case must be verified as caused by a bat RABV variant”. None of the few individuals thought to have survived rabies had documentation of a bat RABV variant [66] [6]. This included a notable 2004 Wisconsin case, with a definite bat bite [66]. The absence of isolated viruses in these cases may be due in part to the impact of an effective host immune response, use of antiviral agents during therapy and lack of postmortem samples.

Twelve cases had removed bats from their dwelling. Only two had known bites. Five of these individuals had both “a bat in the bedroom” and were the person who removed the bats. When bats are reported in homes, instruction on the proper and safe removal of bats to prevent injury, and questioning to determine if any contact with the bat had already occurred, may prevent additional cases. The CDC and others provide guidance on the safe removal of bats from living quarters [67] and prevention for exclusion from living quarters [68]. Most bats do not have rabies. Safe capture and submission for diagnostic testing of bats suspected of human exposure will alleviate unnecessary concern, PEP and associated medical costs.

This review supports current recommendations of the Canadian National Advisory Committee on Immunization (NACI), [3] and the United States Advisory Committee on Immunization Practices (ACIP) [18] related to situations when bats are found in human living quarters, such as bedrooms.

The ACIP recommendations changed from 1999 where “post-exposure prophylaxis can be considered for persons who were in the same room as the bat and who might be unaware that a bite or direct contact had occurred (e.g., a sleeping person awakens to find a bat in the room)” [69] to 2008 where sleep “situations that might qualify as exposures” now include the word deep “(e.g., a deeply sleeping person awakens to find a bat in the room or an adult witnesses a bat in the room with a previously unattended child, mentally disabled person, or intoxicated person)” [18].

The current NACI recommendations state “When a bat is found in the room with a child or an adult who is unable to give a reliable history, assessment of direct contact may be difficult. Factors indicating that direct contact may have occurred include the individual waking up crying or upset while the bat was in the room or observation of an obvious bite or scratch mark.”

Determining whether an exposure to a rabid bat, warranting PEP, has occurred or not, given the diverse “bat-encounter” scenarios a potentially exposed individual confronts, remains a difficult task for both clinicians and public health officials. Adding to this difficulty is the extremely high fatality rate of rabies in humans and both the high costs and the possibility of adverse events associated with PEP. One can easily appreciate the importance of a thorough interview and risk assessment when attempting to gather bat-contact information from any individuals involved in the “bat-encounter” event during the PEP decision-making process.

Both NACI and ACIP recommend that PEP should be given after direct contact when a bite or scratch cannot be ruled out. In addition, NACI specifies that “bat bites may not be felt and leave no visible mark” and “Direct contact with a bat is defined as a bat touching or landing on a person.” Asking about direct contact with claws and teeth may identify exposures that an individual does not consider a bite or scratch but which may result in transmission of RABV infection.

Clearly, effective health messaging and thorough risk assessments related to rabies, bat conservation, management of potential exposures, and the humane, effective exclusion of bats from human dwellings is necessary. As one notable example, during 2011, a woman died of rabies in South Carolina [54]. She had bats in her dwelling and had personally removed bats from her bedroom. No bites were reported. When she contacted a local official for more information about bats in houses and their removal, she was told to not disturb the bats on conservation grounds. However, no discussions about rabies, personal protective equipment, bat-proofing of living spaces, medical consultation, or PEP ensued. She died approximately one month later. A bat RABV was identified and evidence of the presence of bats in the home was documented upon a subsequent public health investigation. This case illustrates the complexity of managing such conflicts at the human-animal interface, in a thorough One Health context, to benefit all species concerns [70].

This synopsis has four primary limitations: 1) the review is based upon published data; 2) the bat exposure event is often brief, and may not be remembered weeks to months later, by the infected individual, given prolonged incubation periods; 3) the diagnosis was frequently not considered while the infected individual was able to be interviewed; and finally, 4) bite marks from bats (unlike typical exposure from carnivores) would not be obvious to the interviewed family members, or colleagues.

The findings in this review underline the importance of identifying those individuals who had an opportunity for UPC with a bat that potentially include the teeth, claws, or saliva, whether in bedrooms, homes, or outside, and not just recognizable bites, scratches, or mucous membrane exposures. Asking individuals about unprotected contact with bat teeth and claws (including a bat landing or crawling on a person) represents an additional measure in helping to determine bat RABV variant exposures.

## Supporting Information

S1 File Systematic Review of Rabies PRISMA 2009 checklist. This is the PLOS ONE required checklist for systematic reviews.
(PDF)

## References

[1] Feder HM, Petersen BW, Robertson KL, Rupprecht CE. Rabies: still a uniformly fatal disease? Historical occurrence, epidemiological trends, and paradigm shifts. Curr. Infect. Dis. Rep. [Internet]. 2012 [cited 2015 Dec 1];14:408–22. Available: http://www.ncbi.nlm.nih.gov/pubmed/22699971 10.1007/s11908-012-0268-2 22699971

[2] Dyer JL, Wallace R, Orciari L, Hightower D, Yager P, Blanton JD. Rabies surveillance in the United States during 2012. J. Am. Vet. Med. Assoc. [Internet]. 2013 [cited 2015 Dec 1];243:805–15. Available: http://www.ncbi.nlm.nih.gov/pubmed/24004227 10.2460/javma.243.6.805 24004227

[3] National Committee on Immunication. Public Health Agency of Canada. Active vaccines, rabies vaccines (part 4) [Internet]. Can. Immun. Guid. Evergr. Ed. Ottawa 2012 [cited 2016 Feb 10]. Available: http://www.phac-aspc.gc.ca/publicat/cig-gci/p04-rabi-rage-eng.php

[4] Middleton D, Johnson KO, Rosatte RC, Hobbs JL, Moore SR, Rosella L, et al Human Rabies Post-Exposure Prophylaxis and Animal Rabies in Ontario, Canada, 2001–2012. Zoonoses Public Health [Internet]. 2015 [cited 2016 Jan 20];62:356–64. Available: http://www.ncbi.nlm.nih.gov/pubmed/25244148 10.1111/zph.12155 25244148

[5] Huot C, De Serres G, Duval B, Maranda-Aubut R, Ouakki M, Skowronski DM. The cost of preventing rabies at any cost: post-exposure prophylaxis for occult bat contact. Vaccine [Internet]. 2008 [cited 2015 Dec 31];26:4446–50. Available: http://www.sciencedirect.com/science/article/pii/S0264410X08008207 10.1016/j.vaccine.2008.06.076 18602958

[6] Monroe BP, Yager P, Blanton J, Birhane MG, Wadhwa A, Orciari L, et al Rabies surveillance in the United States during 2014. J. Am. Vet. Med. Assoc. [Internet]. American Veterinary Medical Association 1931 North Meacham Road—Suite 100, Schaumburg, IL 60173 USA 847-925-8070 847-925-1329 avmajournals@avma.org; 2016 [cited 2016 Mar 25];248:777–88. Available: http://avmajournals.avma.org/doi/abs/10.2460/javma.248.7.777 10.2460/javma.248.7.777 27003019

[7] USGS Report Series Circular 1329: Bat Rabies and Other Lyssavirus Infections [Internet]. [cited 2015 Dec 1]. Available: http://pubs.usgs.gov/circ/circ1329/

[8] Ellison JA, Johnson SR, Kuzmina N, Gilbert A, Carson WC, VerCauteren KC, et al Multidisciplinary approach to epizootiology and pathogenesis of bat rabies viruses in the United States. Zoonoses Public Health [Internet]. 2013 [cited 2015 Dec 2];60:46–57. Available: http://www.ncbi.nlm.nih.gov/pubmed/23137052 10.1111/zph.12019 23137052

[9] Kuzmin I, Rupprecht C. Bat Rabies In: Jackson A, Wunner W, editors. Rabies. 2nd ed London: Academic Press; 2007 p. 259–307.

[10] Patyk K, Turmelle A, Blanton JD, Rupprecht CE. Trends in national surveillance data for bat rabies in the United States: 2001–2009. Vector Borne Zoonotic Dis. [Internet]. 2012 [cited 2015 Dec 2];12:666–73. Available: http://www.ncbi.nlm.nih.gov/pubmed/22607069 10.1089/vbz.2011.0839 22607069

[11] Streicker DG, Turmelle AS, Vonhof MJ, Kuzmin IV., McCracken GF, Rupprecht CE. Host Phylogeny Constrains Cross-Species Emergence and Establishment of Rabies Virus in Bats. Science (80-.). [Internet]. 2010 [cited 2015 Oct 9];329:676–9. Available: http://www.ncbi.nlm.nih.gov/pubmed/20689015 10.1126/science.1188836 20689015

[12] Baer GM. The History of Rabies. Rabies. Elsevier Ltd; 2007 p. 19.

[13] Freuling CM, Klöss D, Schröder R, Kliemt A, Müller T. The WHO Rabies Bulletin Europe: a key source of information on rabies and a pivotal tool for surveillance and epidemiology. Rev. Sci. Tech. [Internet]. 2012 [cited 2015 Dec 2];31:799–807. Available: http://www.ncbi.nlm.nih.gov/pubmed/23520734 23520734 10.20506/rst.31.3.2152

[14] Schatz J, Ohlendorf B, Busse P, Pelz G, Dolch D, Teubner J, et al Twenty years of active bat rabies surveillance in Germany: a detailed analysis and future perspectives. Epidemiol. Infect. [Internet]. 2014 [cited 2015 Dec 2];142:1155–66. Available: http://www.ncbi.nlm.nih.gov/pubmed/24007822 10.1017/S0950268813002185 24007822 PMC9161227

[15] Shwiff SA, Sterner RT, Jay MT, Parikh S, Bellomy A, Meltzer MI, et al Direct and indirect costs of rabies exposure: a retrospective study in southern California (1998–2002). J. Wildl. Dis. [Internet]. 2007 [cited 2015 Dec 2];43:251–7. Available: http://www.ncbi.nlm.nih.gov/pubmed/17495309 17495309 10.7589/0090-3558-43.2.251

[16] Dhankhar P, Vaidya SA, Fishbien DB, Meltzer MI. Cost effectiveness of rabies post exposure prophylaxis in the United States. Vaccine [Internet]. 2008 [cited 2016 Feb 10];26:4251–5. Available: http://www.ncbi.nlm.nih.gov/pubmed/18599167 10.1016/j.vaccine.2008.05.048 18599167

[17] Dato VM, Campagnolo ER, Shah DU, Bellush MJ, Rupprecht CE. Recurrent Temporary Paralysis Reported After Human Rabies Post-Exposure Prophylaxis. Zoonoses Public Heal. 2014/07/06 ed. 2014; 10.1111/zph.12143 24995792

[18] Manning SE, Rupprecht CE, Fishbein D, Hanlon CA, Lumlertdacha B, Guerra M, et al Human rabies prevention—United States, 2008: recommendations of the Advisory Committee on Immunization Practices. MMWR. Recomm. Rep. [Internet]. 2008 [cited 2015 Nov 16];57:1–28. Available: http://www.ncbi.nlm.nih.gov/pubmed/18496505 18496505

[19] Dietzschold B, Morimoto K, Hooper DC, Smith JS, Rupprecht CE, Koprowski H. Genotypic and phenotypic diversity of rabies virus variants involved in human rabies: implications for postexposure prophylaxis. J. Hum. Virol. [Internet]. [cited 2015 Nov 11];3:50–7. Available: http://www.ncbi.nlm.nih.gov/pubmed/10774807 10774807

[20] Krebs JW, Noll HR, Rupprecht CE, Childs JE. Rabies surveillance in the United States during 2001. J. Am. Vet. Med. Assoc. [Internet]. 2002 [cited 2016 Mar 29];221:1690–701. Available: http://www.ncbi.nlm.nih.gov/pubmed/12494966 12494966 10.2460/javma.2002.221.1690

[21] Texas Deparatment of State Health Services. Human Rabies Cases in the United State Attributed to Bat Rabies Variants [Internet]. 2015. Available: http://www.dshs.state.tx.us/idcu/disease/rabies/information/bats/

[22] De Serres G, Dallaire F, Côte M, Skowronski DM. Bat rabies in the United States and Canada from 1950 through 2007: human cases with and without bat contact. Clin. Infect. Dis. 2008;46:1329–37. 10.1086/586745 18419432

[23] Gibbons R V. Cryptogenic rabies, bats, and the question of aerosol transmission. Ann. Emerg. Med. [Internet]. 2002 [cited 2015 Dec 2];39:528–36. Available: http://www.ncbi.nlm.nih.gov/pubmed/11973559 11973559 10.1067/mem.2002.121521

[24] Blanton JD, Dyer J, McBrayer J, Rupprecht CE. Rabies surveillance in the United States during 2011. J. Am. Vet. Med. Assoc. [Internet]. 2012 [cited 2015 Nov 23];241:712–22. Available: http://www.ncbi.nlm.nih.gov/pubmed/22947154 10.2460/javma.241.6.712 22947154 PMC5120402

[25] Centers for Disease Control and Prevention. Human rabies—Texas, 1990. MMWR. Morb. Mortal. Wkly. Rep. [Internet]. 1991 [cited 2015 Nov 16];40:132–3. Available: http://www.ncbi.nlm.nih.gov/pubmed/1900099 1900099

[26] Centers for Disease Control and Prevention. Human rabies—Texas, Arkansas, and Georgia, 1991. MMWR. Morb. Mortal. Wkly. Rep. [Internet]. 1991 [cited 2015 Dec 3];40:765–9. Available: http://www.ncbi.nlm.nih.gov/pubmed/1944123 1944123

[27] Human rabies—New York, 1993. MMWR. Morb. Mortal. Wkly. Rep. [Internet]. 1993 [cited 2016 Mar 31];42:799, 806 Available: http://www.ncbi.nlm.nih.gov/pubmed/8413167 8413167

[28] Human rabies—Texas and California, 1993. MMWR. Morb. Mortal. Wkly. Rep. [Internet]. 1994 [cited 2016 Mar 31];43:93–6. Available: http://www.ncbi.nlm.nih.gov/pubmed/8302264 8302264

[29] Human rabies—California, 1994. MMWR. Morb. Mortal. Wkly. Rep. [Internet]. 1994 [cited 2016 Mar 31];43:455–8. Available: http://www.ncbi.nlm.nih.gov/pubmed/8208235 8208235

[30] Centers for Disease Control and Prevention. Human rabies—West Virginia, 1994. MMWR. Morb. Mortal. Wkly. Rep. [Internet]. 1995 [cited 2015 Dec 3];44:86–7, 93. Available: http://www.ncbi.nlm.nih.gov/pubmed/7838088 7838088

[31] Human rabies—Alabama, Tennessee, and Texas, 1994. MMWR. Morb. Mortal. Wkly. Rep. [Internet]. 1995 [cited 2016 Mar 31];44:269–72. Available: http://www.ncbi.nlm.nih.gov/pubmed/7708035 7708035

[32] Centers for Disease Control and Prevention. Human rabies—Washington, 1995. MMWR. Morb. Mortal. Wkly. Rep. [Internet]. 1995 [cited 2015 Dec 3];44:625–7. Available: http://www.ncbi.nlm.nih.gov/pubmed/7643847 7643847

[33] Centers for Disease Control and Prevention. Human rabies—California, 1995. MMWR. Morb. Mortal. Wkly. Rep. [Internet]. 1996 [cited 2015 Dec 3];45:353–6. Available: http://www.ncbi.nlm.nih.gov/pubmed/8604213 8604213

[34] Human rabies—Connecticut, 1995. MMWR. Morb. Mortal. Wkly. Rep. [Internet]. 1996 [cited 2016 Mar 31];45:207–9. Available: http://www.ncbi.nlm.nih.gov/pubmed/8609874 8609874

[35] Human rabies—Kentucky and Montana 1996. MMWR. Morb. Mortal. Wkly. Rep. [Internet]. 1997 [cited 2016 Mar 31];46:397–400. Available: http://www.ncbi.nlm.nih.gov/pubmed/9157274 9157274

[36] Human rabies—Montana and Washington, 1997. MMWR. Morb. Mortal. Wkly. Rep. [Internet]. 1997 [cited 2015 Dec 8];46:770–4. Available: http://www.ncbi.nlm.nih.gov/pubmed/9272584 9272584

[37] Centers for Disease Control and Prevention. Human rabies—Texas and New Jersey, 1997. MMWR. Morb. Mortal. Wkly. Rep. [Internet]. 1998 [cited 2015 Nov 23];47:1–5. Available: http://www.ncbi.nlm.nih.gov/pubmed/9450721 9450721

[38] Human rabies—Virginia, 1998. MMWR. Morb. Mortal. Wkly. Rep. [Internet]. 1999 [cited 2016 Mar 31];48:95–7. Available: http://www.ncbi.nlm.nih.gov/pubmed/10072266 10072266

[39] Centers for Disease Control and Prevention. Human rabies—California, Georgia, Minnesota, New York, and Wisconsin, 2000. MMWR. Morb. Mortal. Wkly. Rep. [Internet]. 2000 [cited 2015 Dec 3];49:1111–5. Available: http://www.ncbi.nlm.nih.gov/pubmed/11917926 11917926

[40] Human rabies—Iowa, 2002. MMWR. Morb. Mortal. Wkly. Rep. [Internet]. 2003 [cited 2016 Mar 31];52:47–8. Available: http://www.ncbi.nlm.nih.gov/pubmed/12570321 12570321

[41] Human rabies—California, 2002. MMWR. Morb. Mortal. Wkly. Rep. [Internet]. 2002 [cited 2016 Mar 31];51:686–8. Available: http://www.ncbi.nlm.nih.gov/pubmed/12233911 12233911

[42] Human rabies—Tennessee, 2002. MMWR. Morb. Mortal. Wkly. Rep. [Internet]. 2002 [cited 2016 Mar 31];51:828–9. Available: http://www.ncbi.nlm.nih.gov/pubmed/12353742 12353742

[43] Human death associated with bat rabies—California, 2003. MMWR. Morb. Mortal. Wkly. Rep. [Internet]. 2004 [cited 2016 Mar 31];53:33–5. Available: http://www.ncbi.nlm.nih.gov/pubmed/14737062 14737062

[44] Investigation of rabies infections in organ donor and transplant recipients—Alabama, Arkansas, Oklahoma, and Texas, 2004. MMWR. Morb. Mortal. Wkly. Rep. [Internet]. 2004 [cited 2016 Mar 31];53:586–9. Available: http://www.ncbi.nlm.nih.gov/pubmed/15241303 15241303

[45] Srinivasan A, Burton EC, Kuehnert MJ, Rupprecht C, Sutker WL, Ksiazek TG, et al Transmission of Rabies Virus from an Organ Donor to Four Transplant Recipients. N. Engl. J. Med. [Internet]. Massachusetts Medical Society; 2005;352:1103–11. Available: 10.1056/NEJMoa043018 15784663

[46] Blanton JD, Hanlon CA, Rupprecht CE. Rabies surveillance in the United States during 2006. J. Am. Vet. Med. Assoc. [Internet]. American Veterinary Medical Association 1931 North Meacham Road—Suite 100, Schaumburg, IL 60173 USA 847-925-8070 847-925-1329 avmajournals@avma.org; 2007 [cited 2015 Nov 23];231:540–56. Available: http://www.ncbi.nlm.nih.gov/pubmed/17696853 17696853 10.2460/javma.231.4.540

[47] Centers for Disease Control and Prevention. Human rabies—Indiana and California, 2006. MMWR. Morb. Mortal. Wkly. Rep. [Internet]. 2007 [cited 2015 Dec 3];56:361–5. Available: http://www.ncbi.nlm.nih.gov/pubmed/17443120 17443120

[48] Imported human rabies—California, 2008. MMWR. Morb. Mortal. Wkly. Rep. [Internet]. 2009 [cited 2016 Mar 31];58:713–6. Available: http://www.ncbi.nlm.nih.gov/pubmed/19590490 19590490

[49] Human rabies—Missouri, 2008. MMWR. Morb. Mortal. Wkly. Rep. [Internet]. 2009 [cited 2016 Mar 31];58:1207–9. Available: http://www.ncbi.nlm.nih.gov/pubmed/19893481 19893481

[50] Human rabies—Kentucky/Indiana, 2009. MMWR. Morb. Mortal. Wkly. Rep. [Internet]. 2010 [cited 2016 Mar 31];59:393–6. Available: http://www.ncbi.nlm.nih.gov/pubmed/20379132 20379132

[51] Centers for Disease Control and Prevention. Human rabies—Michigan, 2009. MMWR. Morb. Mortal. Wkly. Rep. [Internet]. 2011 [cited 2015 Dec 3];60:437–40. Available: http://www.ncbi.nlm.nih.gov/pubmed/21490561 21490561

[52] Centers for Disease Control and Prevention. Human rabies from exposure to a vampire bat in Mexico—Louisiana, 2010. MMWR. Morb. Mortal. Wkly. Rep. 2011;60:1050–2. 21832976

[53] Human rabies—Wisconsin, 2010. MMWR. Morb. Mortal. Wkly. Rep. [Internet]. 2011 [cited 2016 Mar 31];60:1164–6. Available: http://www.ncbi.nlm.nih.gov/pubmed/21881547 21881547

[54] Centers for Disease Control and Prevention. Human rabies—South Carolina, 2011. MMWR. Morb. Mortal. Wkly. Rep. [Internet]. 2013 [cited 2015 Dec 2];62:642–4. Available: http://www.ncbi.nlm.nih.gov/pubmed/23945770 23945770 PMC4604777

[55] Greer DM, Robbins GK, Lijewski V, Gonzalez RG, Gonzales RG, McGuone D. Case records of the Massachusetts General Hospital. Case 1–2013. A 63-year-old man with paresthesias and difficulty swallowing. N. Engl. J. Med. [Internet]. 2013 [cited 2015 Dec 3];368:172–80. Available: http://www.ncbi.nlm.nih.gov/pubmed/23301735 10.1056/NEJMcpc1209935 23301735

[56] Pratt PD, Henschel K, Turabelidze G, Grim A, Ellison JA, Orciari L, et al Human Rabies—Missouri, 2014. MMWR. Morb. Mortal. Wkly. Rep. [Internet]. 2016 [cited 2016 Mar 21];65:253–6. Available: http://www.cdc.gov/mmwr/volumes/65/wr/mm6510a1.htm?s_cid=mm6510a1_w 10.15585/mmwr.mm6510a1 26985578

[57] Harrist A, Styczynski A, Wynn D, Ansari S, Hopkin J, Rosado-Santos H, et al Human Rabies—Wyoming and Utah, 2015. MMWR. Morb. Mortal. Wkly. Rep. [Internet]. 2016 [cited 2016 Jun 2];65:529–33. Available: http://www.cdc.gov/mmwr/volumes/65/wr/mm6521a1.htm?s_cid=mm6521a1_e#suggestedcitation 10.15585/mmwr.mm6521a1 27253630

[58] Dato VM. 1990–2015, United States of America (the Country), Non-transplant rabies cases with documented bat strains of rabies [Internet]. Apollo Libr. 2016 [cited 2016 Jan 8]. Available: http://epimodels.org/purls/rabiesCaseSeries

[59] Update: investigation of rabies infections in organ donor and transplant recipients—Alabama, Arkansas, Oklahoma, and Texas, 2004. MMWR. Morb. Mortal. Wkly. Rep. [Internet]. 2004 [cited 2016 Apr 5];53:615–6. Available: http://www.ncbi.nlm.nih.gov/pubmed/15254455 15254455

[60] Pape WJ, Fitzsimmons TD, Hoffman RE. Risk for rabies transmission from encounters with bats, Colorado, 1977–1996. Emerg. Infect. Dis. [Internet]. [cited 2016 May 19];5:433–7. Available: http://www.pubmedcentral.nih.gov/articlerender.fcgi?artid=2640787&tool=pmcentrez&rendertype=abstract 10341181 10.3201/eid0503.990315 PMC2640787

[61] CDC—Bats: Learning about bats and rabies—Rabies [Internet]. [cited 2016 May 19]. Available: http://www.cdc.gov/rabies/bats/education/index.html

[62] De Serres G, Skowronski DM, Mimault P, Ouakki M, Maranda-Aubut R, Duval B. Bats in the bedroom, bats in the belfry: reanalysis of the rationale for rabies postexposure prophylaxis. Clin. Infect. Dis. [Internet]. 2009 [cited 2015 Dec 2];48:1493–9. Available: http://www.ncbi.nlm.nih.gov/pubmed/19400689 10.1086/598998 19400689

[63] Oregon Health Authority. Adult Behavior Risk (BRFSS) Results by Topic [Internet]. [cited 2015 Mar 12]. Available: https://public.health.oregon.gov/BirthDeathCertificates/Surveys/AdultBehaviorRisk/Pages/brfsdata.aspx

[64] Authority OH. Oregon Behavioral Risk Factor Surveillance System (BRFSS), 1998 [Internet]. 1998. Available: https://public.health.oregon.gov/BirthDeathCertificates/Surveys/AdultBehaviorRisk/brfssresults/Documents/1998/rabies.pdf

[65] Oregon Behavioral Risk Factor Surveillance System (BRFSS), 1999 [Internet]. 1999. Available: https://public.health.oregon.gov/BirthDeathCertificates/Surveys/AdultBehaviorRisk/brfssresults/Documents/1999/rabies.pdf

[66] Recovery of a patient from clinical rabies—Wisconsin, 2004. MMWR. Morb. Mortal. Wkly. Rep. [Internet]. 2004 [cited 2016 May 29];53:1171–3. Available: http://www.ncbi.nlm.nih.gov/pubmed/15614231 15614231

[67] CDC—Bats: To capture a bat—Rabies [Internet]. [cited 2016 May 20]. Available: http://www.cdc.gov/rabies/bats/contact/capture.html

[68] CDC—Bats: Keeping bats out of your house—Rabies. [cited 2016 May 31]; Available: http://www.cdc.gov/rabies/bats/management/

[69] Centers for Disease Control and Prevention. Human rabies prevention—United States, 1999. Recommendations of the Advisory Committee on Immunization Practices (ACIP). MMWR. Recomm. Rep. [Internet]. 1999 [cited 2015 Dec 2];48:1–21. Available: http://www.ncbi.nlm.nih.gov/pubmed/10077411 10077411

[70] Lankau EW, Cox SW, Ferguson SC, Blanton JD, Tack DM, Petersen BW, et al Community survey of rabies knowledge and exposure to bats in homes—Sumter County, South Carolina, USA. Zoonoses Public Health [Internet]. 2015 [cited 2015 Dec 2];62:190–8. Available: http://www.ncbi.nlm.nih.gov/pubmed/24815566 10.1111/zph.12135 24815566 PMC5774857

